# On the Swallowing of Insoluble Foreign Bodies by Children

**Published:** 1872-05

**Authors:** John H. Packard


### ARTICLE XII.

### *On the Swallowing of Insoluble Foreign Bodies by Children.*

BY JOHN H. PACKARD, M. D.

Notwithstanding the apparently great risk of impaction of the coins, pins, or other insoluble bodies so often swallowed by children, it is very seldom that any serious trouble occurs; probably because the thick mucous lining the intestestinal canal, and the accumulation of fecal matter around the foreign body, prevent the latter from becoming entangled and bear it on with them until it is expelled per anum.

I feel the less called upon to apologize for reporting the following cases, since quite recently the subject was deemed important enough to be discussed in several communications to one of the leading London Journals; the exact reference has unfortunately escaped me.

*Case I.*—September 5, 1868, a child of Mr. S., æt. four months, swallowed an iron box-key. It was one and three-sixteenths of an inch long, and five-eights of an inch wide. Before coming to me, the mother had given the child a teaspoonful of castor oil. I advised letting him alone and watching the evacuations. (They had already begun feeding the child, young as he was, with bread and milk.)

September 7.—At about 8 A. M. the child had a large passage, and the key came away easily, forty-eight hours from the time it was swallowed.

*Case II.*—January 19, 1872, Mr. V's child, æt. ten months, was much troubled by the irritation of a tooth just coming through. The mother was rubbing the gums with a silver safety-pin, when it suddenly slipped into the pharynx and was swallowed. A dose of oil was immediately given, and next day the father came to me. I advised the same course as in the last case; and on the 29th, ten days after the pin disappeared, it was voided by the anus.

The first idea that occurs to the popular mind in such cases always is to give a purgative. The disadvantage of this course, however, in stimulating the bowel to contraction and thereby endangering the entanglement of the foreign body, must be at once evident, and is pointed out by Cooper Forster, Holmes, and others. If the child is old enough to eat, the food given should be such as to produce a large fecal formation.

Several years ago, while visiting a patient, I was called into the next house to see a child who was said to have swallowed a nickel cent. A few days afterwards, inquiring how the child was, I was told that after I had gone away the coin had been found on the floor. The child had dropped it instead of swallowing it.—*Med. Times.*

